# Chloroplast Phylogenomic Inference of Green Algae Relationships

**DOI:** 10.1038/srep20528

**Published:** 2016-02-05

**Authors:** Linhua Sun, Ling Fang, Zhenhua Zhang, Xin Chang, David Penny, Bojian Zhong

**Affiliations:** 1 Jiangsu Key Laboratory for Biodiversity and Biotechnology, College of Life Sciences, Nanjing Normal University, Nanjing, China; 2 Institute of Fundamental Sciences, Massey University, Palmerston North, New Zealand

## Abstract

The green algal phylum Chlorophyta has six diverse classes, but the phylogenetic relationship of the classes within Chlorophyta remains uncertain. In order to better understand the ancient Chlorophyta evolution, we have applied a site pattern sorting method to study compositional heterogeneity and the model fit in the green algal chloroplast genomic data. We show that the fastest-evolving sites are significantly correlated with among-site compositional heterogeneity, and these sites have a much poorer fit to the evolutionary model. Our phylogenomic analyses suggest that the class Chlorophyceae is a monophyletic group, and the classes Ulvophyceae, Trebouxiophyceae and Prasinophyceae are non-monophyletic groups. Our proposed phylogenetic tree of Chlorophyta will offer new insights to investigate ancient green algae evolution, and our analytical framework will provide a useful approach for evaluating and mitigating the potential errors of phylogenomic inferences.

Chloroplast phylogenomics has become a useful approach to elucidate the enigmatic evolutionary relationships of different taxonomic levels of plants^1^,^2^,^3^,^4^. However, resolving the ancient phylogenetic history remains difficult because of non-phylogenetic signal^5^, or use of simplistic substitution models^6^ in the large-scale molecular data. It is known mathematically that the phylogenetic signal fall off exponentially with time at the deepest divergences^7^, thus accurately reconstructing ancient divergence is a difficult task. It has long been suggested that non-phylogenetic signal exist in ancient divergences^8^, and these signal are frequently generated by fast-evolving or compositionally heterogeneous sites^9^,^10^,^11^,^12^. Simplistic substitution models have a poor fit to the data, resulting in un-reliable phylogenetic reconstruction^4^,^13^,^14^. Non-phylogenetic signal and simplistic models are possibly major causes of errors in the large-scale genomic data, and it is critical to reduce the impact of these errors for deep-level phylogenetic reconstruction.

Green algae are estimated to have originated over 1.8 billion years ago^15^, and it splits early into two lineages: the Charophytes and the Chlorophyta^16^,^17^. Resolving the Charophytes phylogeny has received attention because of their close evolutionary relationship to the land plants^12^,^18^,^19^,^20^, but a reliable phylogeny of Chlorophyta is important to understanding ancient evolution and diversification of morphological and cytological characters of green algae.

Chlorophyta comprises six diverse morphological groups that live in both marine and freshwater environments, and includes Prasinophyceae, Ulvophyceae, Trebouxiophyceae, Chlorophyceae, Chlorodendrophyceae and Pedinophyceae^21^,^22^. It has been previously suggested that Ulvophyceae, Trebouxiophyceae and Chlorophyceae are all monophyletic groups, and the common term “UTC clade” defines the grouping of these three classes^23^,^24^,^25^. However, the monophyly of Ulvophyceae and Trebouxiophyceae is not strongly supported based on several molecular studies^22^,^26^,^27^, and the new term “core Chlorophyta” has been recently used to define UTC groups plus Chlorodendrophyceae and Pedinophyceae^21^,^22^. Thus the phylogenetic relationships within Chlorophyta remain controversial. The three phylogenetic topologies between Ulvophyceae, Trebouxiophyceae and Chlorophyceae have been hypothesized: (1) Ulvophyceae be placed as sister to Chlorophyceae^28^,^29^,^30^; (2) Ulvophyceae sister to Trebouxiophyceae^31^,^32^; (3) Chlorophyceae sister to Trebouxiophyceae^33^,^34^.

Here, we attempt to reconstruct a more reliable phylogenetic tree of Chlorophyta both by using a site-heterogeneous substitution model, and by removing non-phylogenetic signal. By estimating the relative evolutionary rate and among-site compositional heterogeneity, we demonstrate that fastest-evolving sites show strong compositional heterogeneity, and have a much poorer fit to the evolutionary model. By removing these fastest-evolving sites and using a site-heterogeneous model, we produced a congruent phylogenetic tree of Chlorophyta supporting the following: (1) The class Chlorophyceae is a monophyletic group. (2) The class Ulvophyceae is a polyphyletic group. (3) The class Trebouxiophyceae is a paraphyletic group. (4) The class Chlorophyceae is likely close to a group uniting Chlorodendrophyceae and one clade of Ulvophyceae.

## Results and Discussion

The original data set included 30,240 nucleotide positions of 53 protein-coding genes from 53 green algae taxa (Supplementary Table S1), and the posterior predictive test indicated that the assumption of compositional homogeneity is strongly violated in the original data (global z-score = 14.52). To reduce the among-lineage compositional heterogeneity of the data set, we excluded the taxa with the most strongly deviating nucleotide composition. The test statistic for individual taxa indicated that the nucleotide composition of 10 taxa is significantly deviating (z-score ranges from 16.10 to 34.68; Supplementary Table S1). When these 10 taxa were excluded from the original data set, the among-lineage compositional heterogeneity is strongly reduced (global z-score = 4.08). Furthermore, two extremely long-branch Trentepohliales (Ulvophyceae) taxa (*Trentepohlia annulata* and *Cephaleuros parasiticus*, see Supplementary Fig. S1) were removed to minimize the potential long-branch attraction artifact^35^ and further prevent the violation of the assumption of compositional homogeneity (global z-score = 3.68). The final data matrix consisted of 53 protein-coding genes from 41 taxa.

**Figure 1:**
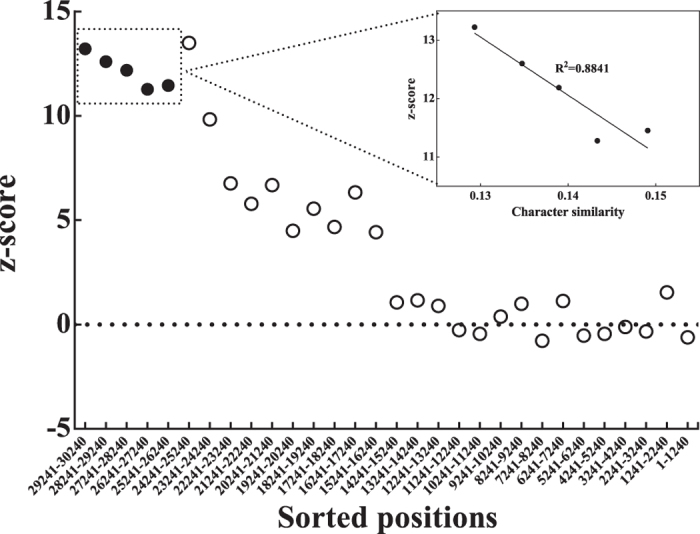
The correlation between the fast-evolving sites and compositional heterogeneity for a series of subsets, each subset having 1,000 sites. The 5,000 fastest-evolving sites are marked with five solid circles. The large z-score value means strong among-site compositional heterogeneity. The “character similarity” is negatively correlated with evolutionary rate i.e. sites that are incompatible with few other sites are considered rapidly evolving. The x-axis shows the sorted positions from the most highly variable to the most conserved.

It has been demonstrated that fastest-evolving sites tend to mask genuine phylogenetic signal, and support an erroneous topology^4^,^36^,^37^. First, we investigated the among-site compositional heterogeneity of the final data matrix. The final 30,240 sites of 41 taxa were sorted from most variable to least variable by using the TIGER method, and the compositional heterogeneity was evaluated for a series of subsets, each subset having 1,000 sites. It has been known that compositional heterogeneity manifests most strongly in fast-evolving sites^38^,^39^. Indeed, our correlation results not only showed that the fast-evolving sites exhibit strongly deviating nucleotide composition, but also the 5,000 fastest-evolving sites are significantly correlated with the most strongly site-compositional heterogeneity (*R*^2^ = 0.88) (Fig. 1).

**Figure 2:**
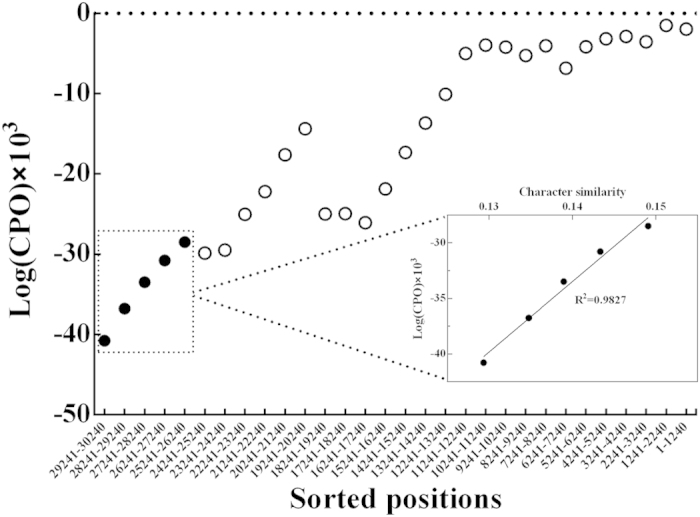
The correlation between the fast-evolving sites and model fitness for a series of subsets, each subset having 1,000 sites. The 5,000 fastest-evolving sites are marked with five solid circles. The log(CPO) values are calculated ranges from -infinity (corresponding to CPO value = 0) to 0 (corresponding to CPO value = 1). The large log(CPO) value means the sites can be accurately predicted by the evolutionary model. The x-axis shows the sorted positions from the most highly variable to the most conserved.

Second, we evaluated the fitness between the substitution model and fast-evolving sites. The Conditional Predictive Ordinates (CPO) method was applied to measure how well data for a site can be predicted by the evolutionary model, and low (and negative) CPO values for the sites indicate the difficulty to predict the site patterns by the model^40^. The CPO analyses showed that the fast-evolving sites have the low CPO values, implying the poor fit to the substitution model. Also, the 5,000 fastest-evolving sites are statistically correlated with the lower CPO values (*R*^2^ = 0.98) (Fig. 2), thus these fastest-evolving sites are not well described by the evolutionary model. There is also a significant correlation between CPO values and compositional heterogeneity for these 5,000 fastest-evolving sites (*R*^2^ = 0.93) (Supplementary Fig. S2), showing that the sites with strongly deviating nucleotide composition have much poorer model fitness.

Often all third codon positions are excluded for phylogenetic analyses because they exhibit higher substitution rates compared to first and second positions. However some third codon positions are relatively slow-evolving and have the genuine phylogenetic signal^41^,^42^, and the first and second codon positions also contain some highly variable sites that should be removed. In our data, there are 3,595 (71.9%) third codon positions and 1,405 (28.1%) first and second positions among the 5,000 fastest-evolving sites. This result shows that not all the third codons are fast-evolving and the site-sorting methods^43^,^44^ are helpful to objectively measure variability at each aligned position.

Because the 5,000 fastest-evolving positions significantly correlate most strongly with compositional heterogeneity, and have a worse fit to the evolutionary model, we produced five shortened data sets (from 29,240 to 25,240 sites) by sequentially removing the 5,000 fastest-evolving sites in 1,000 increments. The cross-validation tests^45^ demonstrated that site-heterogeneous CAT+GTR+Γ model had a much better statistical fit than site-homogeneous GTR+Γ model for these shortened data sets (likelihood score difference: 2,797 ± 82 for 30,240 sites; 2,629 ± 115 for 29,240 sites; 2,417 ± 63 for 28,240 sites; 2,225 ± 92 for 27,240 sites; 1,901 ± 39 for 26,240 sites and 1,682 ± 110 for 25,240 sites, in favor of CAT+GTR+Γ model). We also used other shorter datasets (from 24,240 to 20,240 sites) for reconstructing Chlorophyta phylogeny.

To evaluate the robustness and accuracy of these topologies, we calculated the average bootstrap support values (BSVs) for each inferred topology, and found that the phylogenies with higher BSVs (>90%) are supported based on the data sets ranging from 30,240 to 25,240 sites. Additional shorter data sets supported the topologies but with lower BSVs (<90%), indicating a poor resolution of phylogenetic inference. Thus we focused on the topologies with >90% BSVs (from 29,240 to 25,240 sites) to discuss the green algae relationships.

**Figure 3:**
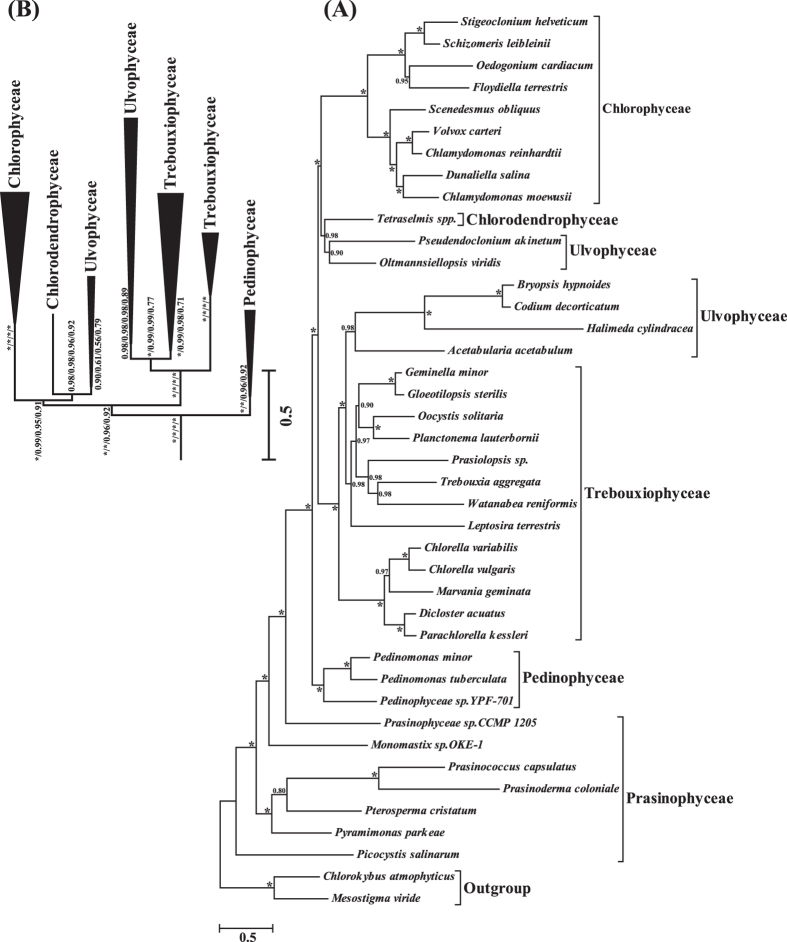
Bayesian phylogenetic trees under the CAT+GTR+Γ site-heterogeneous model. (**A**) The Bayesian tree based on the data set including 30,240 aligned sites from 41 taxa of Chlorophyta. The posterior probability (PP) values are shown on the nodes, and values with full support (1.00 PP) are indicated as*. (**B**) The condensed Bayesian tree of “core Chlorophyta” based on 30,240/29,240/ 28,240/27,240 data sets. The PP values are shown on the nodes from bottom to top corresponding to these 4 data sets, and values with full support (1.00 PP) are indicated as*.

By using a site-heterogeneous CAT+GTR+Γ model and reducing the among-lineage/sites compositional heterogeneity, the Bayesian phylogenetic trees are largely congruent with high support values (Fig. 3; Supplementary Figs S3–S7). They strongly support that Prasinophyceae is paraphyletic, and Prasinophyceae and Pedinophyceae are the early branching lineages of the Chlorophyta. In agreement with recent analyses^22^,^46^,^47^, our phylogenomic analyses show that the class Chlorophyceae is monophyletic with full support (1.00 posterior probability), and the classes Ulvophyceae and Trebouxiophyceae are recovered as polyphyletic and paraphyletic groups respectively. It confirms that the widely accepted term “UTC” clade is invalid and the new term “core Chlorophyta” (previous UTC groups plus the Chlorodendrophyceae and Pedinophyceae) is more appropriate.

In terms of the phylogenetic relationship within Chlorophyta, our chloroplast phylogenomic analyses recovered two separate Ulvophyceae clades. The class Trebouxiopheceae is closest to one Ulvophyceae clade (containing Dasycladales and Bryopsidales), and the class Chlorophyceae is the sister group to a lineage uniting another Ulvophyceae clade (containing Ulotrichales and Oltmannsiellopsidales) and Chlorodendrophyceae (*Tetraselmis*). A notable difference between our phylogeny and recent genome-scale analyses^22^,^46^,^47^ is the position of *Tetraselmis* (Chlorodendrophyceae), *Pseudendoclonium* (Ulotrichales) and *Oltmannsiellopsis* (Oltmannsiellopsidales). *Pseudendoclonium* and *Oltmannsiellopsis* are classified as Ulvophyceae. *Tetraselmis* is a member of the Chlorodendrophyceae and has been reported as an early branching clade of the core Chlorophyta based on nuclear ribosomal data^29^,^48^. However, the recent chloroplast genome-scale analyses recovered *Tetraselmis* is in the vicinity of the *Oltmannsiellopsis*-*Pseudendoclonium* clade^47^ or a clade uniting *Tetraselmis* and *Oltmannsiellopsis* branched early of the core Chlorophyta^22^. Our suggested phylogeny supported *Tetraselmis* is close to *Oltmannsiellopsis*-*Pseudendoclonium* clade, and the *Tetraselmis*-*Oltmannsiellopsis*-*Pseudendoclonium* clade is the sister group to Chlorophyceae. We noted that most of the reported phylogenomic analyses of Chlorophyta have the sparse samples of Ulvophyceae, especially the incomplete sampling of Ulotrichales and Oltmannsiellopsidales. Including more samples from these groups will provide more accurate phylogenetic position of Ulvophyceae.

## Conclusions

By assessing the impact from compositional heterogeneity, fast-evolving sites, and the model fit in the green algal chloroplast genomic data, the correlation analyses show that the sites with fastest evolutionary rates significantly correlate with strong among-site compositional heterogeneity, and have much poorer fit to the evolutionary model. By removing these poor-fitting sites and using a site-heterogeneous substitution model, our chloroplast genomic data consistently indicate that the class Chlorophyceae is a monophyletic group, and the classes Ulvophyceae and Trebouxiophyceae are likely not monophyletic. We further provide a proposed phylogenetic tree of Chlorophyta, although the robust relationship needs more investigations. There are still important uncertainties in phylogenetic relationships within Chlorophyta, and we anticipate that adding more data from Ulvophyceae and Chlorodendrophyceae will help produce a well-supported relationship and make more conclusive inference on the evolution of green algae.

## Materials and Methods

The 53 chloroplast protein-coding genes of 53 taxa (Supplementary Table S1) were collected from three available datasets^22^,^27^,^49^. We first translated the DNA sequences to amino acid using MEGA5^50^, and then aligned them using MUSCLE^51^. Each aligned protein was back-translated to DNA sequence, and was trimmed to exclude poorly aligned positions with complete codons using Gblocks^52^. These alignments were concatenated to generate a matrix of 30,240 sites.

We applied the TIGER method^43^ to estimate the relative evolutionary rate for each site by calculating the pairwise character similarity as a proxy. The “character similarity” is negatively correlated with evolutionary rate i.e. sites that are incompatible with few other sites are considered rapidly evolving. The matrix was then sorted from the most highly varied to the most conserved sites, and a series of subsets (each having 1,000 sites) were generated for further analysis.

To reduce the potential impact of compositional bias in the genome-scale data, we excluded the taxa with the most highly deviating nucleotide composition, and measured the compositional deviation of series of subsets (each having 1,000 sites) by performing the posterior predictive test (z-score as measurement) of compositional homogeneity using the “ppred -comp” command of PhyloBayes^53^. The z-score is the deviation between the observed value of a given test statistic on the original data, and the mean value of the distribution of the test statistic on data replicates under the posterior predictive distribution, divided by the standard deviation of the posterior predictive distribution. A large z-score value indicates the strong compositional heterogeneity.

We evaluated the fitness between the data and evolutionary model using the Conditional Predictive Ordinates (CPO) method^40^ with 100,000 cycles implemented in Phycas program^54^. The CPO provides a posterior predictive approach of how well the individual sites fit the model. The high log(CPO) value means the data can be accurately predicted by the evolutionary model.

We performed a cross-validation test in 10 replicates each with 1,100 cycles to evaluate the relative fit of the site-heterogeneous CAT+GTR+Γ model and the standard site-homogeneous GTR+Γ model on the data sets. The Bayesian phylogenetic trees were reconstructed under the CAT+GTR+Γ model that accounts for site-specific heterogeneity using PhyloBayes^53^. Two independent chains were run for 10,000–20,000 cycles, and the convergence was assessed using the maximum bipartition discrepancies across chains.

## Additional Information

**How to cite this article**: Sun, L. *et al.* Chloroplast Phylogenomic Inference of Green Algae Relationships. *Sci. Rep.* **6**, 20528; doi: 10.1038/srep20528 (2016).

## Supplementary Material

Supplementary Information
