# Supplementary material for: Chloroplast Phylogenomic Inference of Green Algae Relationships
Source: Sci Rep. 2016 Feb 5;6:20528. doi: 10.1038/srep20528 (PMC4742797; doi:10.1038/srep20528)
Supplement: Supplementary Information [file srep20528-s1.pdf]

Chloroplast Phylogenomic Inference of Green Algae Relationships

Linhua Sun[1], Ling Fang[1], Zhenhua Zhang[1], Xin Chang[1], David Penny[2], Bojian Zhong[1,*]

[1]Jiangsu Key Laboratory for Biodiversity and Biotechnology, College of Life Sciences, Nanjing Normal University, Nanjing, China

[2] Institute of Fundamental Sciences, Massey University, Palmerston North, New Zealand

*Corresponding author: Email: bjzhong@gmail.com

Table S1. The list of taxon names and z-scores.

Taxon name	Taxonomy	z-score
Acetabularia acetabulum	Ulvophyceae	8.62
Halimeda cylindracea	Ulvophyceae	6.46
Oltmannsiellopsis viridis	Ulvophyceae	5.94
Trentepohlia annulata	Ulvophyceae	5.66
Bryopsis hypnoides	Ulvophyceae	5.15
Codium decorticatum	Ulvophyceae	4.85
Pseudendoclonium akinetum	Ulvophyceae	3.59
Cephaleuros parasiticus	Ulvophyceae	1.89
Hemichloris antarctica	**Trebouxiophyceae**	**17.36**
Coccomyxa sp.C-169	**Trebouxiophyceae**	**17.16**
Trebouxiophyceae sp.MX-AZ01	**Trebouxiophyceae**	**16.10**
Oocystis solitaria	Trebouxiophyceae	8.69
Geminella minor	Trebouxiophyceae	6.94
Gloeotilopsis sterilis	Trebouxiophyceae	6.36
Planctonema lauterbornii	Trebouxiophyceae	5.50
Parachlorella kessleri	Trebouxiophyceae	4.94
Dicloster acuatus	Trebouxiophyceae	4.83
Leptosira terrestris	Trebouxiophyceae	4.02
Marvania geminata	Trebouxiophyceae	3.14
Trebouxia aggregata	Trebouxiophyceae	3.13
Prasiolopsis sp.	Trebouxiophyceae	2.01
Watanabea reniformis	Trebouxiophyceae	1.40
Chlorella variabilis	Trebouxiophyceae	-0.60
Chlorella vulgaris	Trebouxiophyceae	-0.68
Scenedesmus obliquus	Chlorophyceae	6.66
Oedogonium cardiacum	Chlorophyceae	6.43
Stigeoclonium helveticum	Chlorophyceae	5.62
Schizomeris leibleinii	Chlorophyceae	5.29
Dunaliella salina	Chlorophyceae	5.04
Chlamydomonas moewusii	Chlorophyceae	4.87
Chlamydomonas reinhardtii	Chlorophyceae	1.85
Volvox carteri	Chlorophyceae	1.79
Floydiella terrestris	Chlorophyceae	0.68
Tetraselmis spp.	Chlorodendrophyceae	1.15
Marsupiomonas sp. NIES 1824	**Pedinophyceae**	**32.50**
Pedinomonas tuberculata	Pedinophyceae	2.64
Pedinomonas minor	Pedinophyceae	1.98
Pedinophyceae sp.YPF-701	Pedinophyceae	1.78
Nephroselmis astigmatica	**Prasinophyceae**	**34.68**
Prasinophyceae sp.MBIC 106222	**Prasinophyceae**	**25.32**
Micromonas sp.RCC 299	**Prasinophyceae**	**24.92**
Ostreococcus tauri	**Prasinophyceae**	**24.75**
Nephroselmis olivacea	**Prasinophyceae**	**22.91**
Pycnococcus provasolii	**Prasinophyceae**	**20.60**
Monomastix sp.OKE-1	Prasinophyceae	5.56
Prasinophyceae sp.CCMP 1205	Prasinophyceae	5.36
Prasinoderma coloniale	Prasinophyceae	3.63
Prasinococcus capsulatus	Prasinophyceae	2.90
Pyramimonas parkeae	Prasinophyceae	2.81
Pterosperma cristatum	Prasinophyceae	2.49
Picocystis salinarum	Prasinophyceae	-1.65
Mesostigma viride	Streptophyta	6.21
Chlorokybus atmophyticus	Streptophyta	4.55

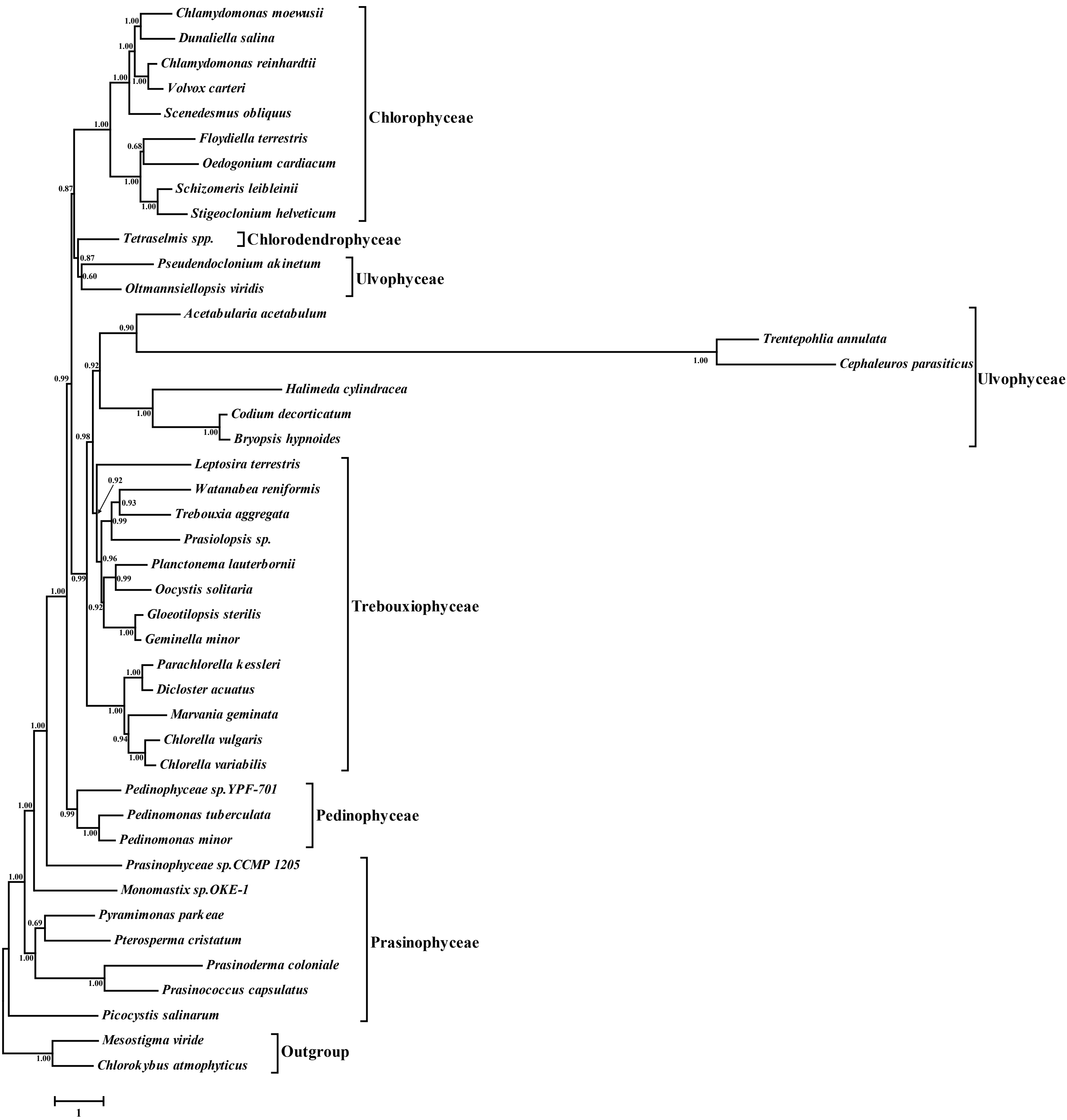

Figure S1. Bayesian phylogenetic tree under the CAT+GTR heterogeneous model based on 30,240 sites with 43 taxa. *Trentepohlia annulata* and *Cephaleuros parasiticus* exhibit extreme long branches. The posterior probability (PP) values are shown on the nodes.

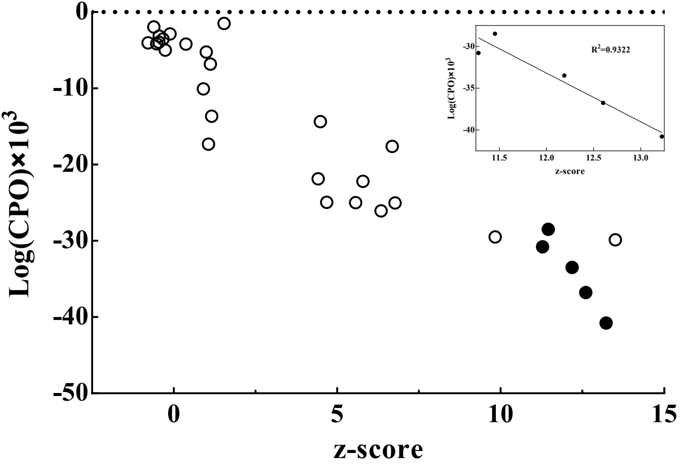

Figure S2. The correlation between the compositional heterogeneity and model fit for a series of subsets, each subset having 1,000 fast-evolving sites. The 5,000 fastest-evolving sites were marked with five solid circles.

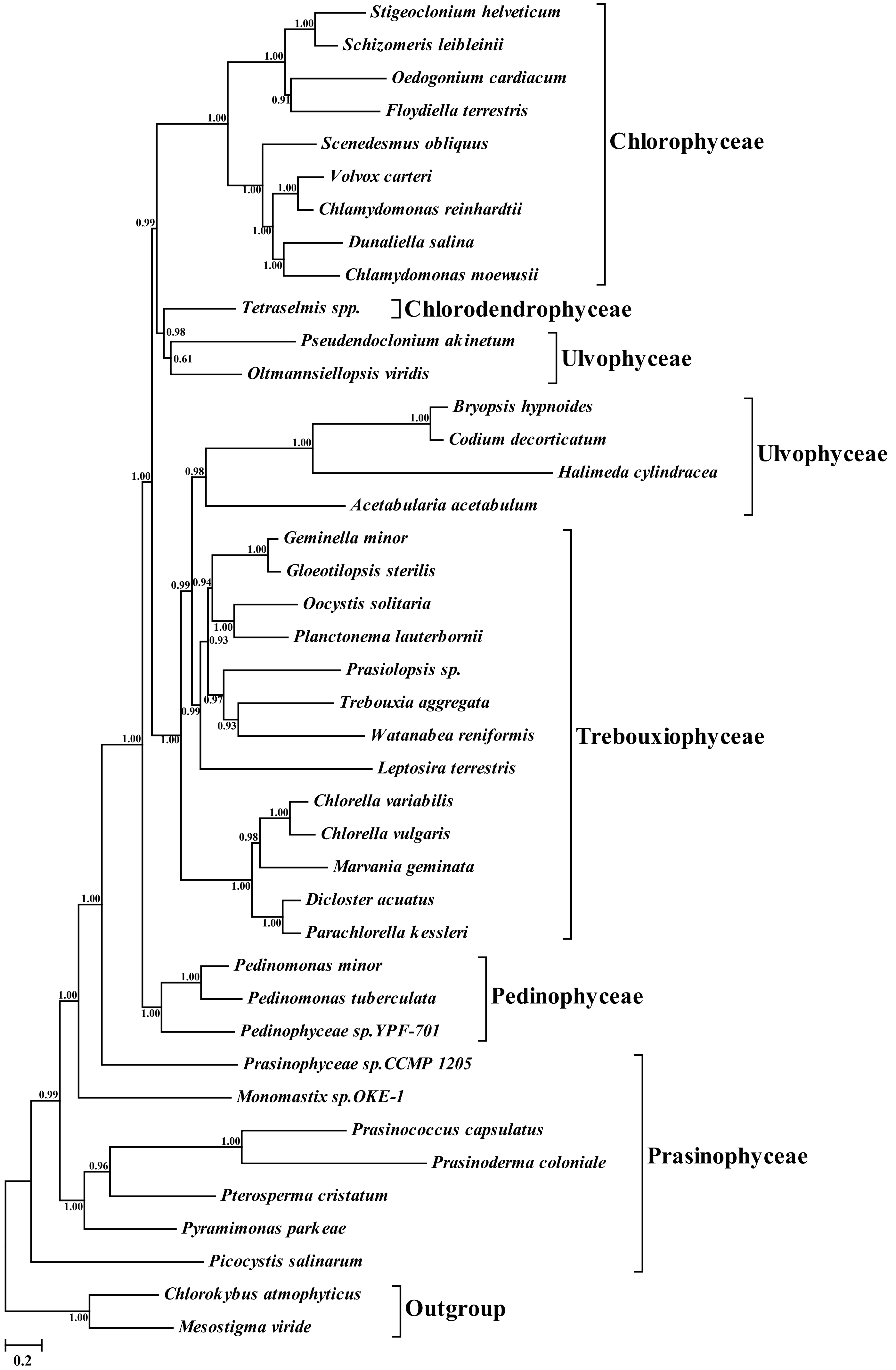

Figure S3. Bayesian phylogenetic tree under the CAT+GTR heterogeneous model based on 29,240 sites with 41 taxa. The posterior probability (PP) values are shown on the nodes.

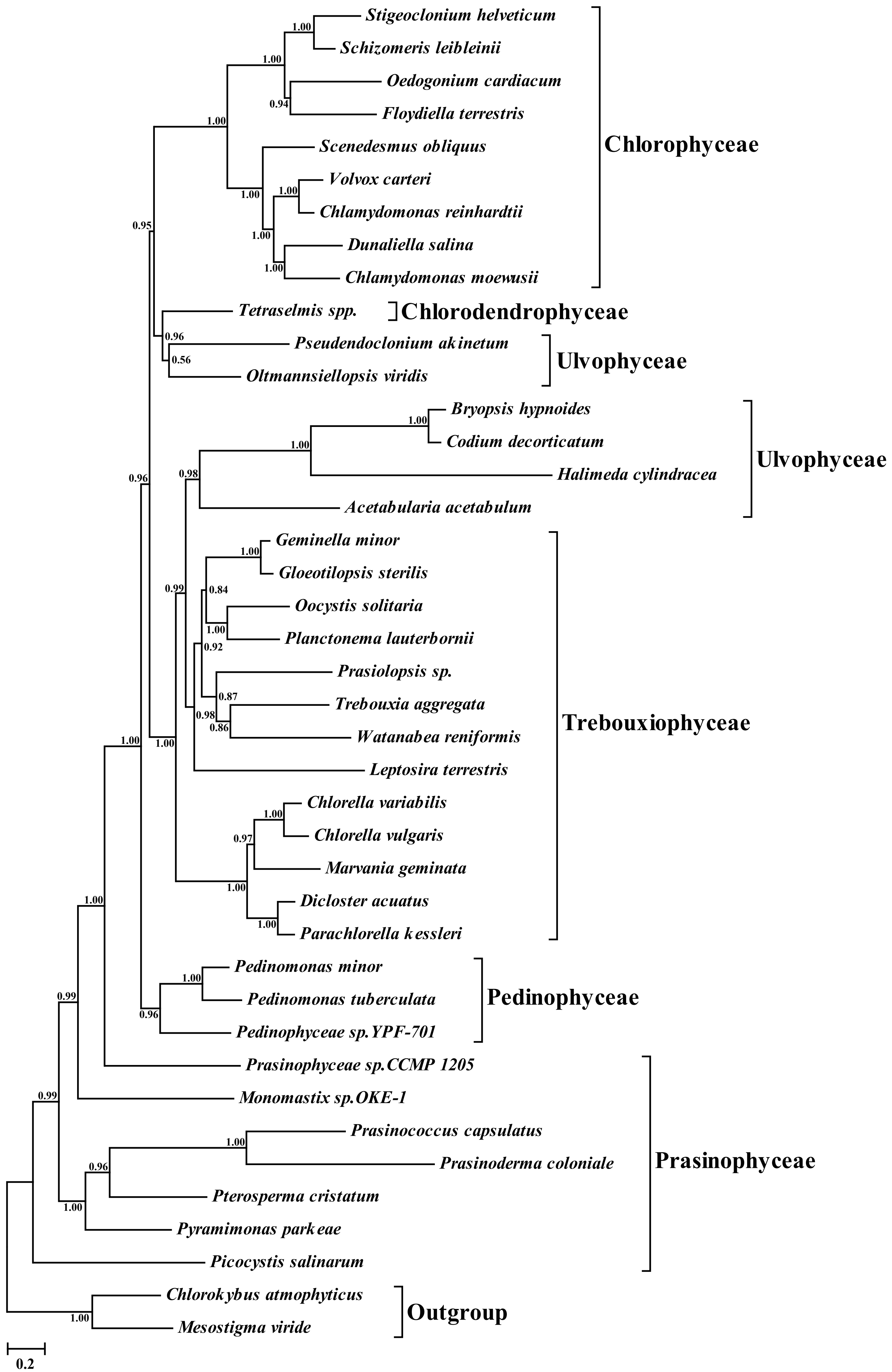

Figure S4. Bayesian phylogenetic tree under the CAT+GTR heterogeneous model based on 28,240 sites with 41 taxa. The posterior probability (PP) values are shown on the nodes.

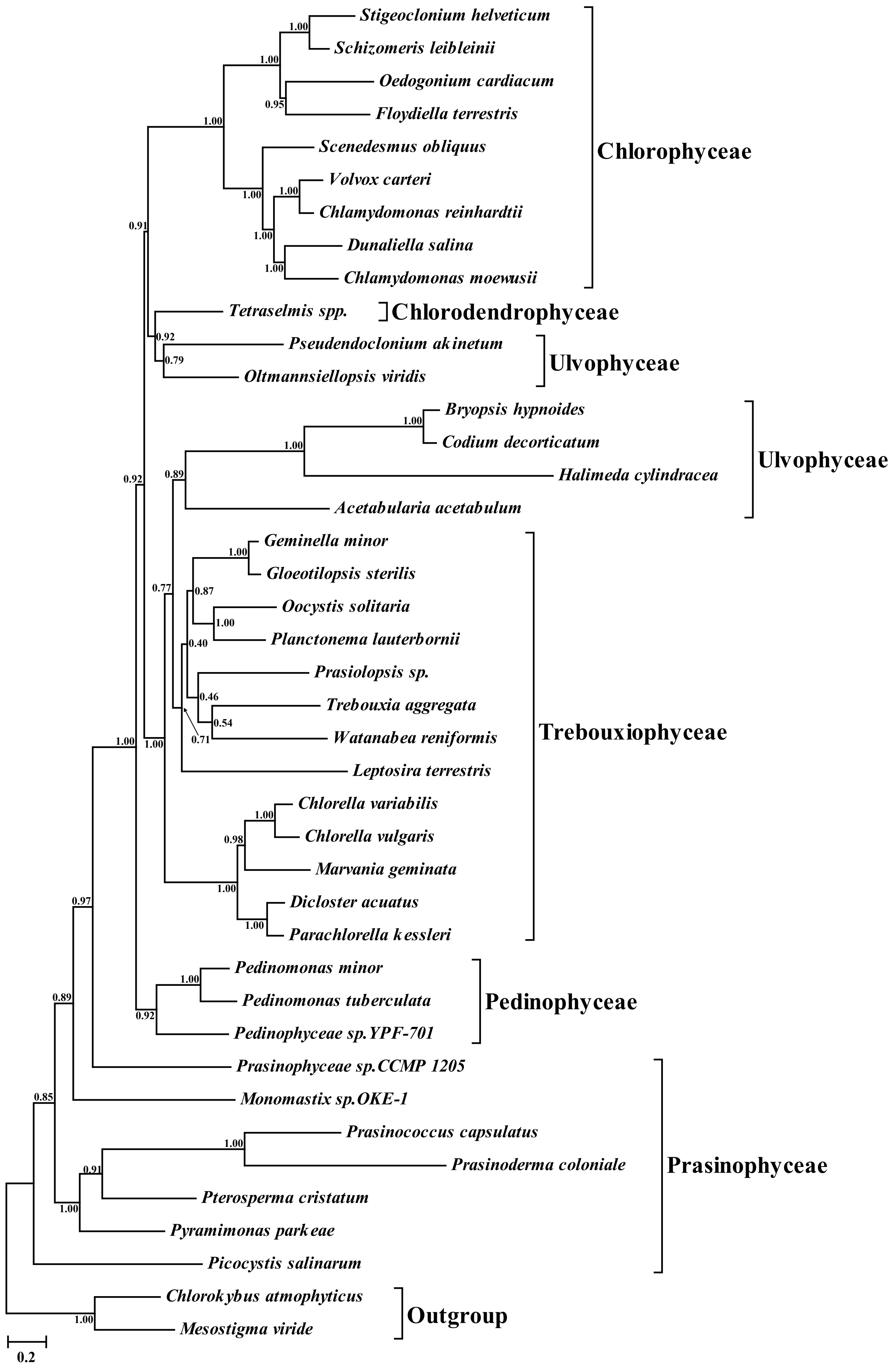

Figure S5. Bayesian phylogenetic tree under the CAT+GTR heterogeneous model based on 27,240 sites with 41 taxa. The posterior probability (PP) values are shown on the nodes.

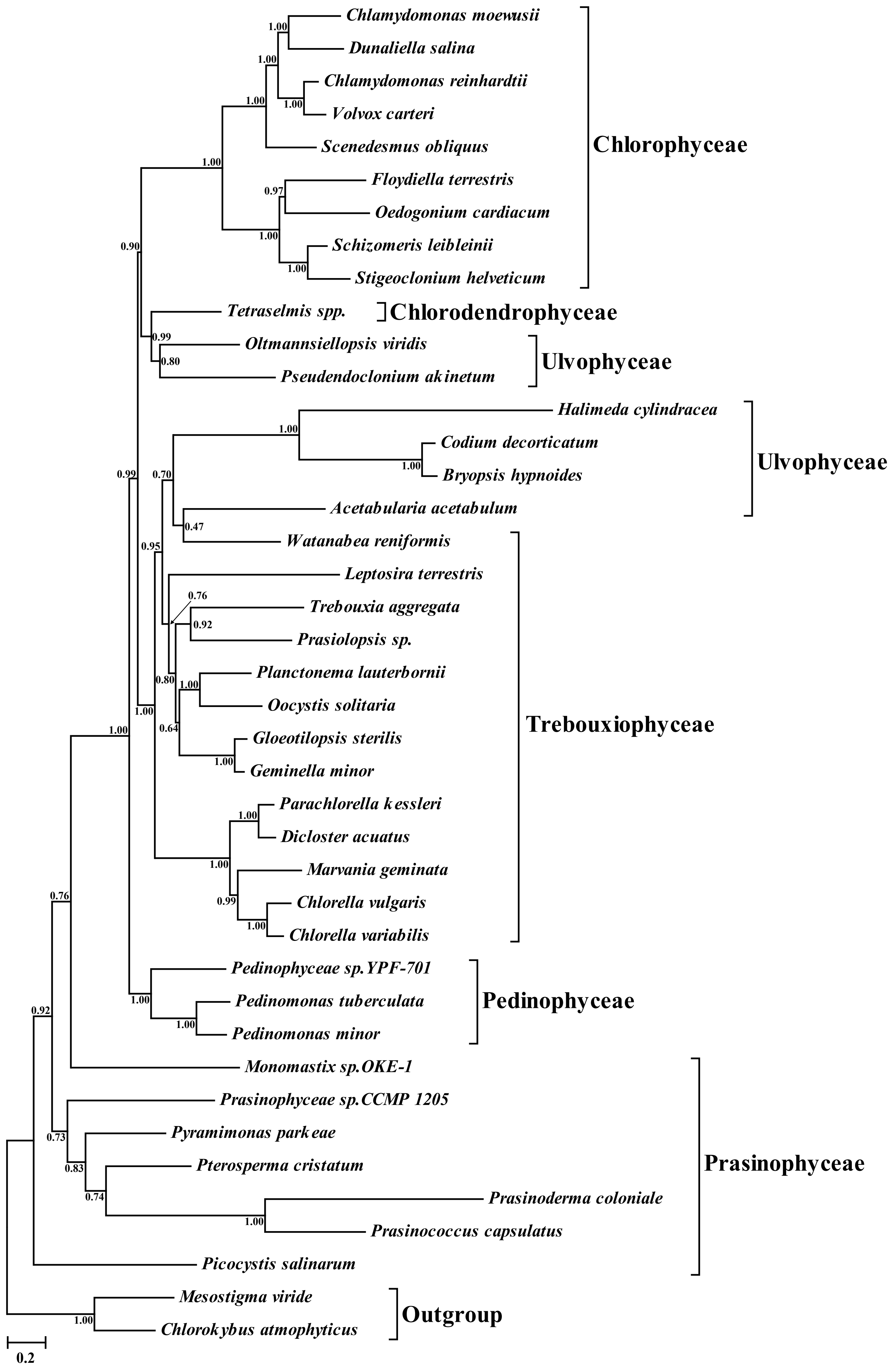

Figure S6. Bayesian phylogenetic tree under the CAT+GTR heterogeneous model based on 26,240 sites with 41 taxa. The posterior probability (PP) values are shown on the nodes.

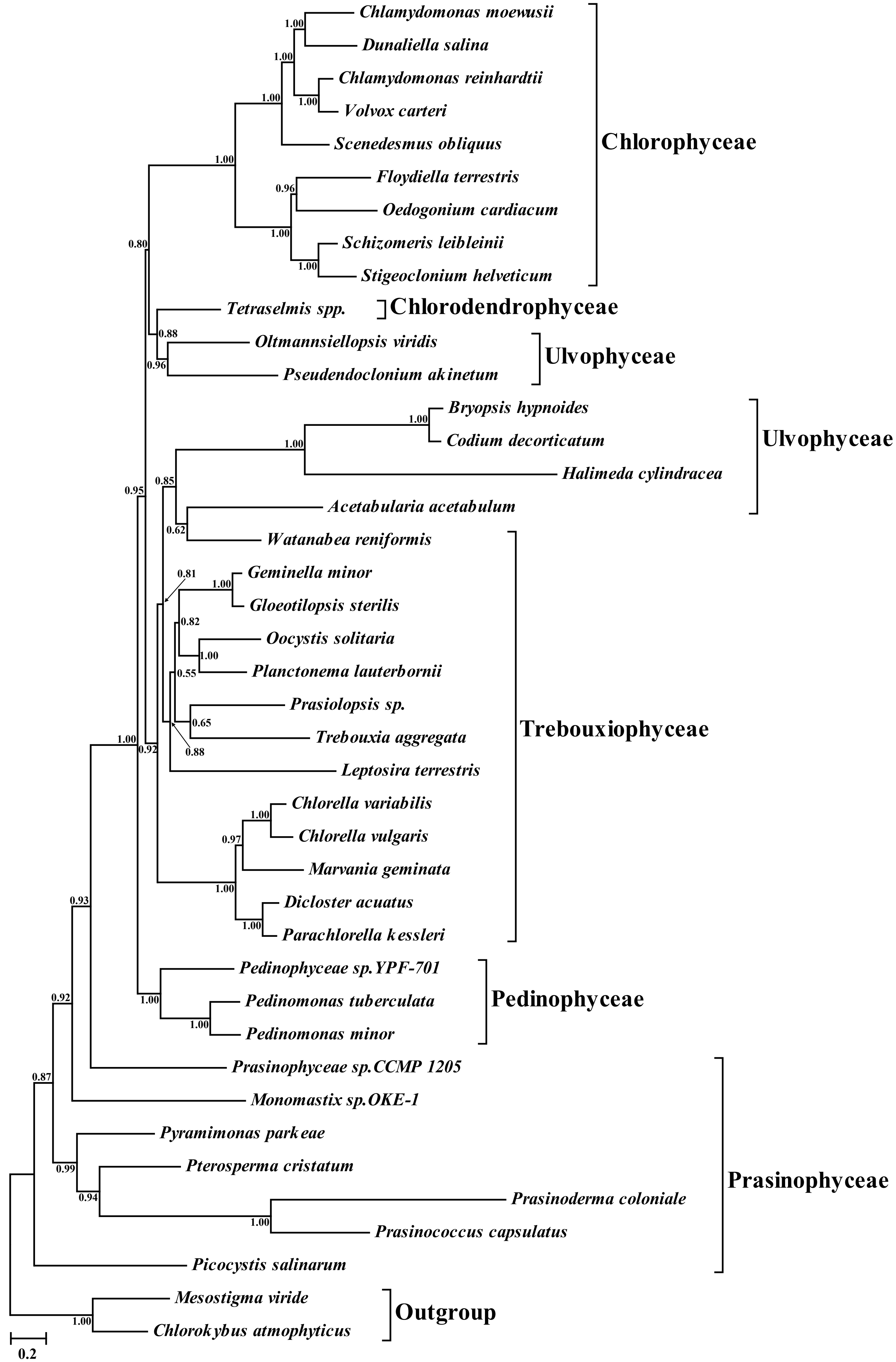

Figure S7. Bayesian phylogenetic tree under the CAT+GTR heterogeneous model based on 25,240 sites with 41 taxa. The posterior probability (PP) values are shown on the nodes.
